# *Armillaria* Species: Biological Complexity, Bioactive Metabolites and Molecular Foundations for Medicinal and Agricultural Applications

**DOI:** 10.3390/biology15120954

**Published:** 2026-06-18

**Authors:** Yingce Duan, Zhenzhu Huang, Xuezhen Yang, Qing Tian, Lei Ye, Bo Zhang, Xiaolin Li

**Affiliations:** 1 Sichuan Institute of Edible Fungi, Sichuan Academy of Agricultural Sciences, Chengdu 610066, China; yingceduan@163.com (Y.D.); huangzhenzhu123@scsaas.cn (Z.H.); yangxz1986@scsaas.cn (X.Y.); tianqing@scsaas.cn (Q.T.); yeray@scsaas.cn (L.Y.); zhangbo1987@scsaas.cn (B.Z.); 2 Key Laboratory of Horticultural Crops Biology and Germplasm Enhancement in Southwest, Ministry of Agriculture and Rural Affairs, Chengdu 610066, China; 3 Environment-Friendly and Efficient Water-Saving Technology and Equipment for Hilly Agriculture Key Laboratory of Sichuan Province, Chengdu 610066, China

**Keywords:** *Armillaria*, nutritional value, protoilludane-type sesquiterpenes, polysaccharides

## Abstract

*Armillaria* is a genus of macrofungi with high ecological, biological, medicinal, and edible value. As facultative plant pathogens and nutritional symbionts, *Armillaria* species support the growth of valuable medicinal plants including *Gastrodia elata* and *Polyporus umbellatus*. They also exhibit unique traits such as exceptional longevity, widespread clonal expansion, rhizomorph formation, and bioluminescence, making them a valuable model for studying fungal ecology, symbiosis, specialized metabolism, and applied research. This review summarizes recent progress in *Armillaria* research, covering biological characteristics, nutritional components, bioactive constituents, species identification, genomic resources, and biosynthetic pathways. We discuss advances in artificial cultivation and the regulatory roles of exogenous phytohormones in mycelial and rhizomorph development. The nutritional value of fruiting bodies is highlighted, with a focus on key pharmacologically active metabolites such as protoilludane-type sesquiterpenes and polysaccharides. We also review multilocus phylogenetic analysis, comparative genomics, and the biosynthetic gene clusters of melleolides and bioluminescence, which have improved understanding of *Armillaria* evolution and functional differentiation.

## Simple Summary

*Armillaria* is a vital edible and medicinal macrofungus with unparalleled worldwide distribution and huge individual biomass compared with other macrofungi. This paper systematically reviews the biological characteristics, bioactive metabolites and molecular regulatory basis of *Armillaria*, and concludes mainstream research hotspots in this field. Specifically, core bioactive substances including secondary metabolites and polysaccharides are separately synthesized in its mycelia and fruiting bodies, and the biosynthetic mechanisms underlying these compounds have attracted extensive research attention. Meanwhile, prominent research gaps remain to be addressed: the biological characteristics of *Armillaria* have not been comprehensively elucidated. Furthermore, only a handful of studies have reported the food-oriented development and utilization of *Armillaria*. Exploring novel food exploitation modes for *Armillaria* species represents a critical research priority for future studies. This review intends to provide novel insights for subsequent in-depth basic research and multi-scenario application of *Armillaria* in medicine and agriculture.

## 1. Introduction

**Figure 1:**
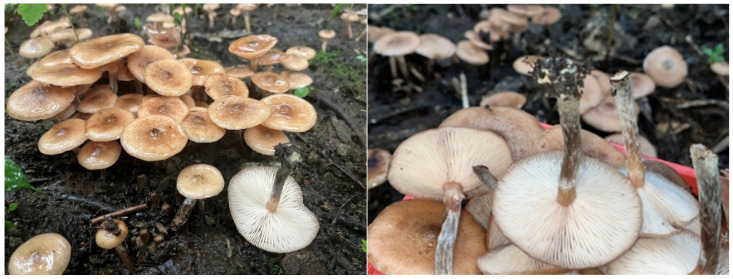
Fruiting body of *Armillaria* sp.

*Armillaria* is a genus of macrofungi classified within the family Physalacriaceae, order Agaricales, and phylum Basidiomycota [1]. This genus is recognized as a significant source of both medicinal and edible fungi, possessing unique nutritional and therapeutic properties. As facultative parasitic fungi, species within the *Armillaria* genus are capable of forming specialized structures known as rhizomorphs during their growth phase (Figure 1). These rhizomorphs facilitate host infection, nutrient absorption, protection of mycelial growth, and expansion of the fungi’s ecological range. Furthermore, *Armillaria* plays a crucial role as a nutritional symbiont in the cultivation of traditional Chinese medicinal plants, such as *Gastrodia elata* [2,3] and *Polyporus umbellatus* [4].

The *Armillaria* genus encompasses a diverse array of species, with notable edible varieties including *A. gallica* (commonly referred to as French honey fungus), *A. ostoyae*, *A. mellea*, *A. borealis*, and *A. luteo-virens*, among others. *Armillaria* is characterized by five key biological traits: extensive growth potential, prolonged lifespan, the ability to produce specialized rhizomorphs, bioluminescence, and reported nitrogen-fixing capabilities [5]. The life cycle of *Armillaria* is complete, commencing with the germination of spores into mycelia, which subsequently differentiate into rhizomorphs. The rhizomorphs infiltrate wood to extract nutrients, and the accumulation of these nutrients is crucial for the development of fruiting bodies. As a distinctive nutritional structure of *Armillaria*, rhizomorphs bear resemblance to plant roots or octopus tentacles and possess the ability to penetrate the rigid xylem of plants, a rare characteristic among macrofungi.

*Armillaria* not only supplies energy to symbiotic organisms such as *G. elata* and *P. umbellatus*, but also facilitates independent reproduction, thereby holding both ecological and economic significance. This paper provides a comprehensive review of the biological characteristics, nutritional quality, active components, and pharmacological effects of *Armillaria*, with the objective of offering a reference for the advanced research, development, and utilization of fungi within the genus *Armillaria*.

## 2. Biological Characteristics of *Armillaria* spp.

Currently, the artificially cultivable species of *Armillaria* that have been documented include *A. gallica*, *A. ostoyae*, and *A. mellea* (commonly known as the hazel mushroom). *A. mellea* is capable of growing within a temperature range of 10 to 33 °C, with an optimal growth temperature of 23 °C and an ideal pH level of 6.0 [6]. Research conducted by Shi et al. identified that the optimal growth medium for the *A. gallica* strain DJ1 is potato dextrose agar supplemented with sawdust and wheat bran. The incorporation of corn flour was found to enhance fruiting, with a medium containing 4% corn flour resulting in the highest fruiting efficiency and the most rapid primordium formation [7]. According to Chen et al., *A. ostoyae* can completely colonize cultivation bags within a period of two months. Fruiting was observed at a temperature of 18 °C, with 60% humidity and 12 h of diffused light; primordia appeared after approximately 10 days. Subsequently, when the humidity in the cultivation environment was increased to 90–95%, yellow fruiting bodies covered with brown scales emerged within 2–3 days [8]. Few published reports are available on the fruiting bodies induction of *Armillaria* under artificial cultivation, highlighting the critical roles of temperature, light, moisture and ventilation in strain domestication. Most commercially available *Armillaria* fruiting bodies are harvested from wild habitats or semi-wild imitative cultivation systems. Accordingly, domestication of more *Armillaria* strains is of great significance for their further development and utilization.

**Figure 2:**
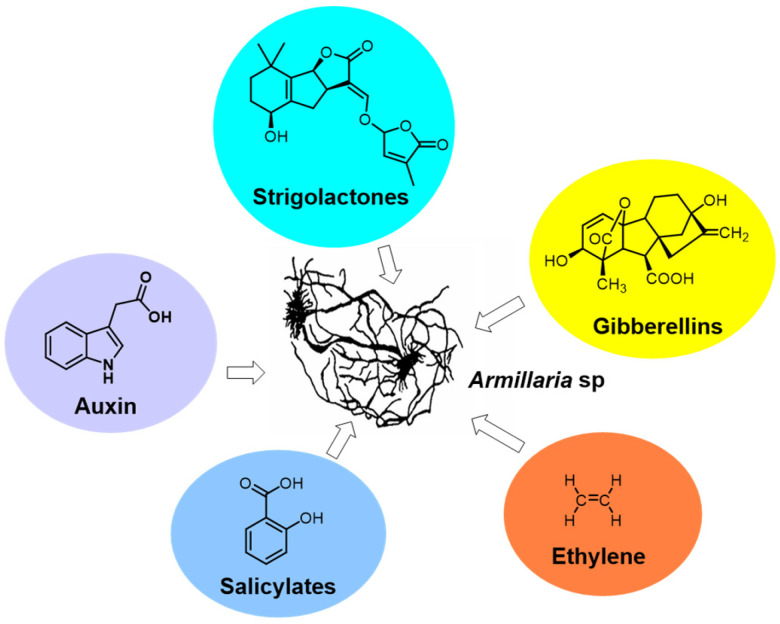
Five major plant hormones have an effect on *Armillaria* spp.

*Armillaria* is a facultatively parasitic fungus characterized by two primary growth forms: mycelia and rhizomorphs. This organism frequently forms symbiotic associations with various plant species, including *G. elata*. More than 40 species have been identified in the genus *Armillaria*, and these fungi are capable of infecting over 500 plant species [9]. These findings also illustrate the intricate growth interactions between plants and *Armillaria*. In order to investigate the exogenous factors that facilitate the growth of *Armillaria*, plant hormones were selected as the experimental additives. Among the nine major classes of plant hormones, approximately five were identified as enhancers of *Armillaria*’s mycelial growth (Figure 2). Importantly, the stimulatory effects of different plant hormones on both mycelial and rhizomorph development are species-specific and dependent on concentration, with their regulatory effects detailed in Table 1.

Cao et al. reported that auxins, including indole-3-acetic acid and indole-3-butyric acid, promoted the mycelial growth of *A. gallica* on agar plates and identified the corresponding differentially expressed genes [10]. They further demonstrated that gibberellins at various concentrations also enhanced mycelial growth, and screened related differentially expressed genes through transcriptomic analysis [11]. In addition, ethephon treatment promoted mycelial growth, and transcriptomic data revealed the presence of ethylene receptors in *A. gallica*, thereby providing new insights into its symbiotic interaction with *G. elata* [12]. Strigolactones synthesized and released by *G. elata* have been detected at the interface between *G. elata* and *Armillaria* rhizomorphs. Treatment with 1 mg/L of the strigolactone analogue GR24 induced branching in *A. gallica* [13]. Strigolactones have also been shown to promote the mycelial growth of several *Armillaria* species. Specifically, supplementation with GR24 at 2.0 μg/L stimulated branching in *A. mellea*, resulting in increased mycelial pellet diameter in liquid culture, the highest number of rhizomorph branches and branching ratio, and elevated mycelial dry weight and polysaccharide content [14]. GR24 at 0.1 µg/mL also promoted rhizomorph bifurcation in *A. gallica* strain 541 [15]. Yang et al. reported that 5.0 μg/mL naphthalene acetic acid, a synthetic plant growth regulator, enhanced both mycelial and rhizomorph growth in *Armillaria* [16]. Zhou et al. found that the plant growth regulator triacontanol significantly accelerated mycelial growth and shortened the cultivation period of *A. luteo-virens* [17]. Luo et al. further revealed that abietic acid, a plant-derived diterpenoid, promoted rhizomorph growth in *A. gallica* [18].

**Table 1:** Exogenous plant hormone addition promotes the growth of *Armillaria* spp. mycelium and rhizomorphs.

Species	Exogenous Additives	Concentration	Reference
*Armillaria gallica*	indole-3-acetic acid	2 mg/L	[10,19]
*Armillaria gallica*	indole-3-butyric acid	2 mg/L	[10]
*Armillaria gallica*	salicylic acid	3.20 ng/mL	[20]
*Armillaria gallica*	naphthalene acetic acid	8 mg/L	[21]
*Armillaria gallica*	gibberellic acid	2 mg/L	[11]
*Armillaria gallica*	ethylene	2 mg/L	[12]
*Armillaria gallica*	abietic acid	0.6 g/L	[18]
*Armillaria gallica*	strigolactones	0.1 µg/mL	[15]
*Armillaria mellea*	strigolactones	2 µg/L	[14]
*Armillaria mellea*	naphthalene acetic acid	5 µg/mL	[16]
*Armillaria luteo-virens*	triacontanol	0.15 ng/mL	[17]

## 3. Nutritional Components of *Armillaria* spp.

The fruiting bodies of *Armillaria* spp. are highly palatable and nutritionally rich, constituting a premium food resource characterized by high protein content, low fat content, and abundant vitamins, dietary fibre, minerals, and diverse polysaccharides. As a widely consumed edible mushroom, commonly known as the hazel mushroom and frequently used in the traditional Chinese dish “Braised Chicken with Mushrooms”, it is highly appreciated for its fleshy texture and distinctive flavour. In terms of nutritional composition, the carbohydrate content of the fruiting bodies reaches 81.25 g/100 g dry weight, whereas the contents of crude protein, crude fat, and ash are 1.81 g/100 g, 1.97 g/100 g, and 8.84 g/100 g, respectively. In addition, mannitol is the principal free sugar, malic acid is the predominant organic acid, and the tocopherol content reaches 42.41 μg/100 g [22]. *Armillaria* is also rich in vitamins, including vitamin B_1_ (8%), vitamin B_2_ (52.50%), vitamin C (11.23%), and vitamin PP (34.15%), as well as trace elements such as selenium and manganese. These constituents confer antioxidant, anti-inflammatory, and potential anticancer properties [23]. Studies have further shown that *A. mellea* from Morocco contain various phenolic acids, such as vanillic acid (198.4 µg/g dw), cinnamic acid (100.6 µg/g dw), protocatechuic acid (48.34 µg/g dw), and gallic acid (32.24 µg/g dw), which are closely associated with antioxidant activity [24]. Vondruska et al. reported that wild-collected fruiting bodies of *A. ostoyae* contained silver (5.6 mg·kg^−1^), calcium (150 mg·kg^−1^), copper (28 mg·kg^−1^), iron (190 mg·kg^−1^), magnesium (1100 mg·kg^−1^), manganese (30 mg·kg^−1^) and zinc (40 mg·kg^−1^). These data confirm that wild *Armillaria* fruiting bodies are safe for edible consumption without excessive heavy metal contamination [25].

## 4. Active Components of *Armillaria* sp. and Their Pharmacological Effects

The medicinal value of *Armillaria* is primarily attributable to its rich repertoire of secondary metabolites, among which protoilludane-type sesquiterpenes and polysaccharides represent the principal bioactive constituents. These metabolites display a broad spectrum of pharmacological activities, thereby providing the material basis for the medicinal exploitation of *Armillaria*.

### 4.1. Protoilludane-Type Sesquiterpenes

**Figure 3:**
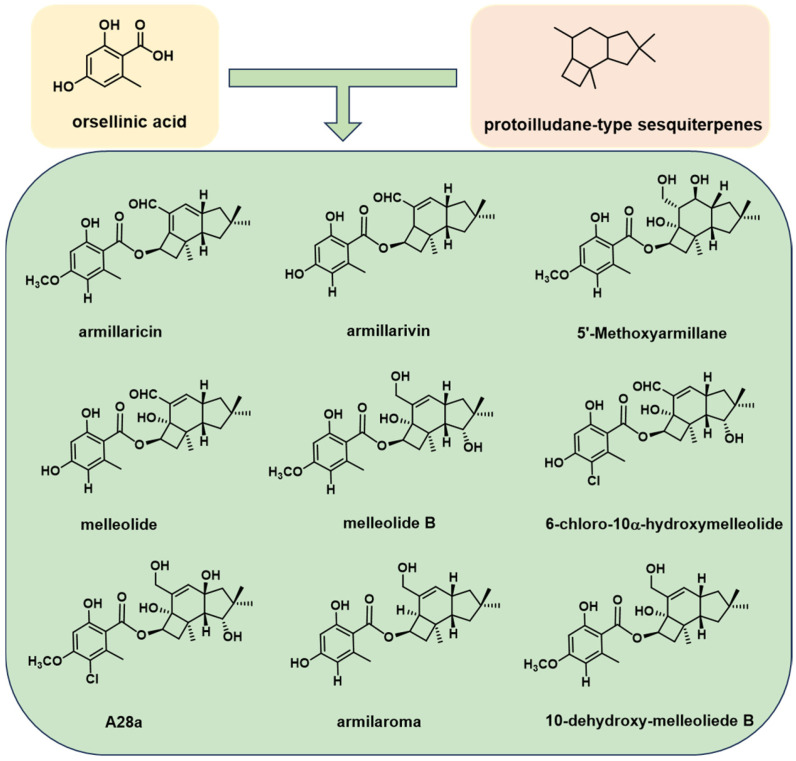
Representative compounds formed by the combination of protoilludane-type sesquiterpenes and orsellinic acid skeletons.

Protoilludane-type sesquiterpenes are widely distributed within the genus *Armillaria*, particularly during the mycelial stage, and have been identified in multiple *Armillaria* species. These compounds are collectively referred to as armillarins. Their core structure typically consists of a protoilludane-type sesquiterpene scaffold linked to an orsellinic acid moiety, and many armillarins undergo further post-skeletal modifications (Figure 3). To date, more than 70 compounds of this class have been reported from the genus *Armillaria* [26]. These metabolites have been documented in a range of *Armillaria* species and are predominantly isolated from mycelia, indirectly suggesting that sesquiterpene synthase expression occurs mainly during the mycelial stage [27]. Armillaricin was isolated from *A. mellea* [28], whereas armillarivin was identified in *A. gallica* [29]; both compounds exhibit cytotoxic activity. Melleolide has been reported in many *Armillaria* species, particularly in *A. tabescens* [30], and represents an important cytotoxic metabolite from which a series of structurally related compounds can be derived. Melleolides constitute a structurally diverse class of polyketide–sesquiterpene hybrids and interfere with fungal translation through elongation factor 2 [31]. 5′-Methoxyarmillane, isolated from the mycelia of *A. ostoyae*, exhibits a wide range of biological activities [32], including antibacterial, antifungal, cytotoxic, and antiviral effects. In addition, compounds such as melleolide B have also been identified in *A. borealis* [33,34]. 6′-Chloro-10α-hydroxymelleolide, isolated from the mycelia of *A. novae-zelandiae*, possesses both antibacterial and antifungal activities [35]. A28a, which is a compound identified in *A. cepistipes*, also exhibits antibiotic activity [36]. Several other sesquiterpene compounds have likewise been reported in the genus *Armillaria*, including armilaroma [37] and 10-dehydroxy-melleolide B [38], both of which show cytotoxic activity. The types and pharmacological effects of protoilludane-type sesquiterpenes identified from different *Armillaria* species are summarized in Table 2.

### 4.2. Polysaccharide

The genus *Armillaria* contains a diverse array of polysaccharides that exhibit a broad spectrum of pharmacological activities, including antioxidant, antibacterial, immunomodulatory, and anticancer effects. Most studies on *Armillaria* polysaccharides have focused on those isolated from fruiting bodies, whereas comparatively fewer have examined polysaccharides derived from mycelia (Table 3). Among these, AMP, a polysaccharide isolated from *A. mellea*, has been reported to possess anticancer activity. AMP exerts potent inhibitory effects on the growth of A549 cells, induces cell-cycle arrest at the G0/G1 phase, and is accompanied by an increased proportion of apoptotic cells [39]. In cyclophosphamide-induced immunosuppressed mice, treatment with the acidic polysaccharide AMPA elevated the levels of several immune-related factors, including IL-2, IL-6, IgM, IgA, TNF-α, and IFN-γ [40].

AMPS-III, a polysaccharide produced by mycelial fermentation, contains the relatively uncommon monosaccharide xylose, suppresses the release of tumor necrosis factor-α, and also exhibits anti-inflammatory activity [41]. AgP, a polysaccharide isolated from *A. gallica*, protects HepG2 cells against hydrogen peroxide-induced oxidative damage, regulates apoptosis-related proteins, and alleviates ROS-mediated cellular apoptosis, thereby demonstrating marked antioxidant activity [42]. ALP-A from *A. luteo-virens* effectively inhibited the growth of S180 solid tumors, protected immune organs, and promoted cytokine secretion [43]. Polysaccharides from *A. tabescens* enhance the proliferative capacity of T cells, B cells, and RAW 264.7 cells, thereby exerting immunomodulatory effects [44].

AT-W, a polysaccharide isolated from the mycelium of *A. tabescens*, comprises several monosaccharide components, including arabinose and trehalose. Notably, AT-W not only reduces serum uric acid levels by inhibiting xanthine oxidase activity, upregulating the expression of ABCG2 and OAT1, and downregulating the expression of URAT1, but also significantly attenuates hyperuricemia-induced renal injury. These findings suggest that AT-W holds considerable therapeutic potential for the treatment of hyperuricemia [45].

### 4.3. Other Active Components

**Figure 4:**
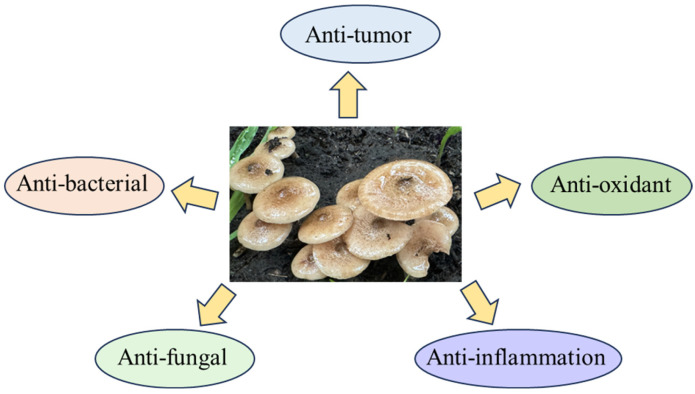
Overview of the pharmacological properties of *Armillaria* spp.

*Armillaria* species also contain various other bioactive constituents, including triterpenes, sterols, lectins, pigments and proteinaceous metabolites. These compounds endow *Armillaria* with diverse biological functions (Figure 4). A dimeric lectin with a molecular weight of 29.4 kDa was isolated from the dried fruiting bodies of *A. luteo-virens*. This lectin is characterized by a novel N-terminal sequence, moderate thermal stability, acid and alkali stability, potent mitogenic activity toward splenocytes, and antiproliferative activity against tumour cells [46]. *A. mellea* also contains triterpenes and sterols, among which the triterpenes are mainly specific pentacyclic triterpenoids, including friedelin and 3-hydroxyfriedel-2-one, whereas the sterols are primarily ergosterol and ergosterol peroxide [47]. In addition, 27.98 g·L^−1^ of melanin was isolated from the fruiting bodies of *A. cepistipes*, and this pigment exhibited antioxidant activity through free-radical scavenging [48].

Co-culture of *A. solidipes* mycelium with *Bacillus velezensis* BY6 was found to inhibit and disrupt the bacterial cell wall and cell membrane, thereby limiting intracellular nutrient utilization in BY6 cells [49]. Furthermore, a multifunctional enzyme named melleatin was identified from the fruiting bodies of *A. mellea*. This molecule exhibits antibacterial membrane activity and antifungal activity against *Trichoderma harzianum* and *Botrytis cinerea* by targeting fungal ribosomes [50]. The ethyl acetate extract of *A. mellea* also exerts anti-inflammatory effects and contains seven compounds, including vanillic acid, syringate, 5-hydroxymethylfurfural, 2-furoic acid, 4-hydroxybenzoic acid, daidzein, and genistein [51]. Studies on chemical constituents of *Armillaria* lay a foundation for species identification and functional research.

## 5. Identification of *Armillaria* Species

The identification of *Armillaria* species is inherently complex and cannot be reliably achieved using internal transcribed spacer (ITS) sequencing alone. Instead, accurate taxonomic resolution requires the combined use of multiple conserved gene regions, as summarized in Table 4. Phylogenetic trees constructed using translation elongation factor 1-alpha (*tef1-α*) sequences from global isolates have been adopted to establish the worldwide phylogenetic framework for the genus [52], and this approach has become a widely accepted and important method for the species identification of *Armillaria*.

Brazee et al. used partial *tef1-α*, RNA polymerase subunit II (*rpb2*), and rDNA large subunit (LSU) sequences to identify isolates representing *A. calvescens* and *A. gallica* in North America [53]. *Tef1-α* and ITS markers are commonly employed to identify *Armillaria* species in Japan [54] and South Korea [55]. A multilocus study further analysed 144 sequences derived from three DNA regions commonly used for fungal identification, namely the ribosomal intergenic spacer 1 (IGS-1) and ITS regions and *tef1-α*, across 48 specimens representing six European *Armillaria* species: *A. cepistipes*, *A. ostoyae*, *A. gallica*, *A. borealis*, *A. mellea*, and *A. tabescens* [56]. *Armillaria* isolates from Serbia were identified using LSU-IGS1 and *tef1-α* sequences [57]. Koch reconstructed the phylogenetic relationships of the *Armillaria*–*Desarmillaria*–*Guyanagaster* lineage using the 28S rRNA, *tef1-α*, RPB2, *β-tubulin*, *actin-1* and glyceraldehyde-3-phosphate dehydrogenase gene sequences. The results revealed that the genus *Armillaria* diverged latest in this lineage. Unlike the other two genera, it evolved the characteristic melanized rhizomorphs [58].

Guo et al. conducted phylogenetic analyses of Chinese *Armillaria* samples using sequences from ITS, *tef1-α*, and *β-tubulin* gene, revealing at least 15 phylogenetic lineages in China, seven of which are associated with cultivated *Gastrodia elata* [59]. Hou et al. described *A. desertorum*, a new species of *Armillaria* (Agaricales, Basidiomycota) from Inner Mongolia, China, based on ITS, LSU, and *tef1-α* sequences combined with morphological and molecular phylogenetic analyses [60]. Qin et al. constructed a phylogenetic species tree from concatenated sequences of *actin*, *h3h*, *hisps*, LSU rDNA, *rpb1*, and *tef1-α*, and identified eight species: *A. algida*, *A. amygdalispora*, *A. bruneocystidia*, *A. luteopileata*, *A. pungentisquamosa*, *A. sinensis*, *A. tibetica*, and *A. violacea* [61]. Klopfenstein et al. further classified *Armillaria* species across the Northern Hemisphere using elongation factor gene sequences [62]. In terms of the identification of Chinese *Armillaria* strains, multi-locus analysis combining multiple conserved gene regions delivers more accurate species delimitation compared with single-locus identification.

**Table 4:** Reference sequences employed for the phylogenetic tree of *Armillaria* classification.

Regions	Sequences	References
North America	partial *tef1-α*, RPB2, and nLSU	[53]
Japan	ITS and *tef1-α*	[54]
South Korea	ITS and *tef1-α*	[55]
Europe	ribosomal IGS-1; ITS; *tef1-α*	[56]
Serbia	LSU-IGS1; *tef1-α*	[57]
China	ITS; *tef1-α*; *β-tubulin*	[59]
China	ITS, LSU and *tef1-α*	[60]
China	*actin*, *h3h*, *hisps*, LSU rDNA, *rpb1*, and *tef1-α*	[61]
Northern Hemisphere	*Tef1-α*	[62]

## 6. Genome of *Armillaria* spp.

To date, a total of 29 *Armillaria* genome sequences had been deposited in the National Center for Biotechnology Information (NCBI) and Joint Genome Institute (JGI) databases. The genome sizes of fungi within the genus *Armillaria* vary substantially, ranging from 40.6 to 102 megabases (Mb). Compared with other related species, this genus exhibits a relatively wide range of genome sizes. After removal of redundant entries, these datasets encompass 17 *Armillaria* species. Detailed information on the sequenced genomes is summarized in Table 5. Nevertheless, all *Armillaria* species share two core functional gene clusters—the melleolide biosynthetic gene cluster and the bioluminescence gene cluster—which represent key diagnostic features of the genus.

## 7. Protoilludane-Type Sesquiterpene Biosynthetic Gene Cluster

**Figure 5:**
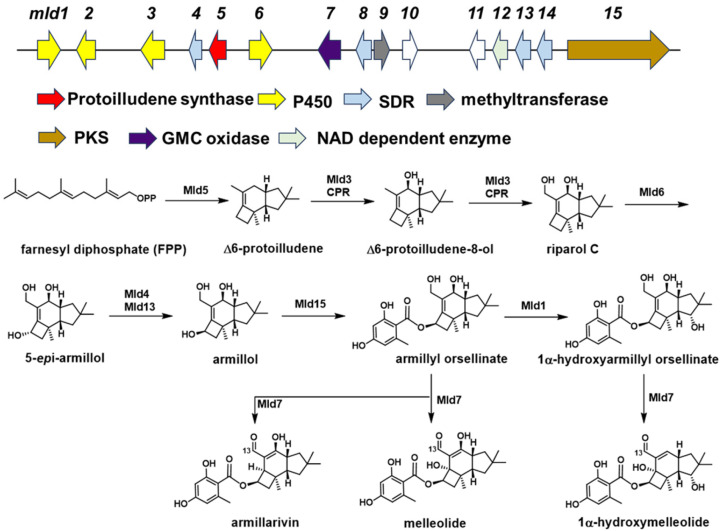
Biosynthetic studies of sesquiterpenoids in *A. mellea*.

Melleolides are characteristic metabolites synthesized by all *Armillaria* species, and their production is primarily governed by the corresponding biosynthetic gene cluster (Figure 5) [72]. The *mld* gene cluster comprises 15 genes. In our survey of 12 *Armillaria* species in the JGI database, this cluster was detected in all species examined, indicating that melleolide biosynthesis is closely associated with the presence of this conserved genetic locus. Using farnesyl pyrophosphate (FPP), generated through the mevalonate pathway, as the precursor substrate, the sesquiterpene synthase Mld5 in *A. mellea* catalyses the cyclization of FPP to produce protoilludane-type sesquiterpenes. Following this step, adjacent P450 enzymes mediate downstream tailoring reactions, while a polyketide synthase (orsellinic acid synthase) Mld15 catalyses the coupling of the orsellinic acid moiety with the protoilludane-type sesquiterpene scaffold. Subsequent modifications mediated by P450 enzymes and GMC oxidoreductases ultimately give rise to the final melleolide products [72]. Elucidation of biosynthetic pathways enables identification of additional intermediate compounds, facilitating the production and development of such compounds via heterologous expression in host strains including *Saccharomyces cerevisiae* and *Aspergillus oryzae*.

## 8. Bioluminescence-Related Gene Cluster

**Figure 6:**
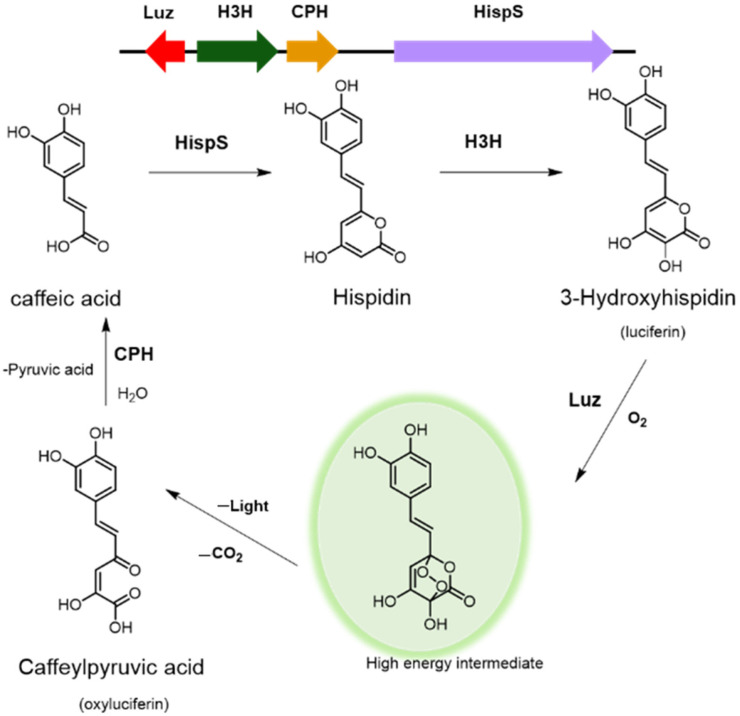
Studies on the bioluminescence mechanism of *Armillaria* spp.

Nearly all *Armillaria* species exhibit bioluminescent properties. The luminescence mechanism uses caffeic acid, derived from the phenylalanine and tyrosine metabolic pathways, as the substrate. Hispidin synthase (HispS) catalyses the formation of hispidin, which is subsequently 3-hydroxylated by 3-hydroxylase (H3H) to generate 3-hydroxyhispidin. Under the action of luciferase (Luz) and molecular oxygen, 3-hydroxyhispidin then emits light while forming a high-energy intermediate containing an epoxide bridge, which ultimately rearranges into caffeylpyruvic acid. Caffeylpyruvic acid is subsequently hydrolysed by caffeylpyruvate hydrolase (CPH), thereby regenerating caffeic acid and completing the luminescence cycle [73]. This biosynthetic gene cluster is present in many bioluminescent mushrooms, including *Neonothopanus gardneri* and *Panellus stipticus* (Figure 6) [73]. In *Armillaria*, it appears to be stably conserved and has been identified in all species for which genome sequences are currently available, further supporting the conclusion that the genus *Armillaria* belongs to the lineage of bioluminescent fungi. Mihail et al. reported luminescence dynamics of different *Armillaria* species to reflect differences in ecological context [74].

## 9. Conclusions

The genus *Armillaria* represents a remarkable fungal lineage at the intersection of ecology, agriculture, medicine and biotechnology. Its biological distinctiveness—manifested in rhizomorph formation, symbiotic competence, plant pathogenicity, bioluminescence and broad environmental adaptability—has made this genus both scientifically compelling and practically valuable. At the same time, its rich nutritional composition and diverse bioactive metabolites, especially protoilludane-type sesquiterpenes and polysaccharides, provide a strong foundation for the development of functional foods, pharmaceuticals and other high-value fungal products.

Recent studies have substantially expanded our understanding of *Armillaria* cultivation, phytohormone-responsive growth regulation, metabolite diversity, species delimitation and genome evolution. *Armillaria* exhibits multiple growth forms, including mycelium, rhizomorphs and fruiting bodies. In commercial cultivation, it is generally maintained in the mycelial and rhizomorph stages to supply nutrients to *G*. *elata* and *P*. *umbellatus*. If macrofungi are continuously subcultured and propagated while remaining in the mycelial phase for a long time, their mycelial activity will decline [75], strains will degenerate, and rhizomorph formation will be inhibited in *Armillaria*. Accordingly, timely induction of fruiting bodies formation to complete the life cycle can effectively restore and enhance the vitality of *Armillaria* mycelium and rhizomorphs. Inducing fruiting in *Armillaria* can rejuvenate its mycelia and yield nutrient-rich fruiting bodies, which represents a promising direction for our future research and development. The above results also demonstrate that studying the biological characteristics of *Armillaria* is of great significance.

In particular, the increasing availability of genomic resources, together with the identification of conserved gene clusters associated with melleolide biosynthesis and fungal bioluminescence, has opened new avenues for linking genotype to phenotype and for elucidating the molecular basis of key adaptive and metabolic traits. Nevertheless, the field continues to face several major bottlenecks, including persistent taxonomic ambiguity, uneven genomic coverage across species, limited functional validation of biosynthetic pathways, and an incomplete mechanistic understanding of host interaction and rhizomorph development.

## Figures and Tables

**Table 2:** Sesquiterpene arylester natural products in the mycelium of *Armillaria* spp.

Species	Compounds	Pharmacological Activity	References
*Armillaria mellea*	armillaricin	cytotoxicity	[28]
*Armillaria gallica*	armillarivin	cytotoxicity	[29]
*Armillaria tabescens*	melleolide	cytotoxicity	[30]
*Armillaria ostoyae*	5′-methoxyarmillane	antibacterial, antifungal, cytotoxic and antiviral	[32]
*Armillaria borealis*	melleolide B	antibacterial activity	[33,34]
*Armillaria novae-zelandiae*	6′-chloro-10α-hydroxymelleolide	antibacterial and antifungal activity	[35]
*Armillaria cepistipes*	A28a	antibiotic activity	[36]
*Armillaria* sp.	armilaroma	cytotoxicity	[37]
*Armillaria* sp.	10-dehydroxy-melleoliede B	cytotoxicity	[38]

**Table 3:** Polysaccharides in the *Armillaria* species: contents, sources, molecular weights, components and pharmacological activities (- means no data in the reference).

Species	Name	Source	MW (kDa)	Monosaccharide Composition	Biological Activity	References
*Armillaria mellea*	AMP	fruiting bodies	460	D-glucose	antitumor	[39]
*Armillaria mellea*	AMPA	fruiting bodies	7.323	Mannose; glucose; galactose	immunomodulatory	[40]
*Armillaria mellea*	AMPS-III	mycelia	13	Galactose; xylose; fucose	anti-inflammation	[41]
*Armillaria gallica*	AgP	fruiting bodies	-	Mannose; Glucose; Galactose	antioxidant	[42]
*Armillaria luteo-virens*	ALP-A	fruiting bodies	23.693	Glucose; mannose	antitumor	[43]
*Armillaria tabescens*	AT-P	fruiting bodies	17.6	Glucose; galactose	immunomodulatory	[44]
*Armillaria tabescens*	AT-W	mycelium	25.6	mannose, galactose, arabinose, and fucose	anti-hyperuricemic effect	[45]

**Table 5:** Statistics of genomic sequencing of *Armillaria* spp.

Number	Species	Strains	Genome Size	Database	References
1	*Armillaria gallica*	M3	86.1 Mb	NCBI	[63]
2	*Armillaria gallica*	012m	87.3 Mb	NCBI	[64]
3	*Armillaria gallica*	NIFoS 2319	86 Mb	NCBI	
4	*Armillaria gallica*	Ar21-2	85.3 Mb	NCBI/JGI	[65]
5	*Armillaria gallica*	Jzi34	79.2 Mb	NCBI	[66]
6	*Armillaria ostoyae*	C18/9	56.7 Mb	NCBI	[65]
7	*Armillaria ostoyae*	C18/9	60.1 Mb	NCBI/JGI	[65]
8	*Armillaria ostoyae*	gfArmOsto1.1	66.1 Mb	NCBI	
9	*Armillaria ostoyae*	gfArmOsto1.1	62.4 Mb	NCBI	
10	*Armillaria solidipes*	ID001	55.7 Mb	NCBI	[67]
11	*Armillaria solidipes*	4-28	58 Mb	NCBI/JGI	[65]
12	*Armillaria solidipes*	GN1	56.6 Mb	NCBI	[68]
13	*Armillaria borealis*	FPL87.14	71.7 Mb	NCBI/JGI	[5]
14	*Armillaria borealis*	AB13-TR4-IP16	66.6 Mb	NCBI	[69]
15	*Armillaria mellea*	ELDO17	70.9 Mb	NCBI/JGI	[5]
16	*Armillaria mellea*	DSM 3731	79.5 Mb	JGI	[70]
17	*Armillaria* sp.	CMW 4456	55 Mb	NCBI	
18	*Armillaria fuscipes*	CMW2740	53 Mb	NCBI	
19	*Armillaria altimontana*	837-10	73.7 Mb	NCBI	[67]
20	*Armillaria calvescens*	YAFA0618	92.2 Mb	NCBI	[71]
21	*Armillaria cepistipes*	B5	75.5 Mb	NCBI/JGI	[65]
22	*Armillaria novae-zelandiae*	ICMP 16352	79.3 Mb	NCBI/JGI	[5]
23	*Armillaria nabsnona*	CMW6904	62.7 Mb	NCBI/JGI	
24	*Armillaria fumosa*	CBS 122221	55.8 Mb	NCBI/JGI	[5]
25	*Armillaria luteobubalina*	HWK02	97.1 Mb	NCBI/JGI	[5]
26	*Armillaria sinapina*	YAFMHJ89	102 Mb	NCBI	
27	*Armillaria mexicana*	IZTA4595	49 Mb	NCBI	[1]
28	*Armillaria tabescens*	CCBAS 213 v1.0	74.8 Mb	JGI	[5]
29	*Armillaria ectypa*	FPL83.16 v1.0	40.6 Mb	JGI	

## Data Availability

Data sharing is not applicable. No new data were created or analyzed in this study.
